# In-service training policy during the COVID-19 pandemic: The case of the agents of the farmers, rural people, and nomads social insurance fund

**DOI:** 10.3389/fpubh.2023.1098646

**Published:** 2023-02-02

**Authors:** Hamed Ghadermarzi, Pouria Ataei, Afshin Mottaghi Dastenaei, Caglar Bassullu

**Affiliations:** ^1^Department of Geography and Rural Planning, Faculty of Geographical Sciences, Kharazmi University, Tehran, Iran; ^2^Department of Agricultural Extension and Education, Faculty of Agriculture, Tarbiat Modares University (TMU), Tehran, Iran; ^3^Department of Political Geography, Faculty of Geographical Sciences, Kharazmi University, Tehran, Iran; ^4^Research Scholar in School of Forest, Fisheries and Geomatics Sciences, University of Florida, Gainesville, FL, United States

**Keywords:** employees' capabilities, farmers, rural people, and nomads social insurance, COVID-19 pandemic, in-service training, job performance

## Abstract

**Introduction:**

The COVID-19 pandemic has posed the in-service training of agents of the Farmers, Rural People, Nomads Social Insurance Fund (hereafter the Fund) to many problems. In-service training is one of the most effective development factors for organizational goals. This sort of training will increase employees' skills and subsequently improve their job performance. Accordingly, the present research mainly aimed to shed light on the effect of in-service training policy on employees' capabilities and job performance.

**Methods:**

The research was conducted among the agents of the Fund in Fars province, Iran (*N* = 197) out of whom 127 agents were sampled by simple randomization. The research instrument was a standard questionnaire whose face and content validity was confirmed by a panel of experts and its reliability was determined by calculating Cronbach's alpha in a pilot study. The results showed that the indicators used to measure the research variables were acceptably consistent with the factor structure and the theoretical framework of the research.

**Results and discussion:**

Based on the findings, in-service training in the Fund during the COVID-19 pandemic has had a positive and significant effect on the agents' capabilities (communication and team-working skills, creativity and problem-solving skills, commitment and responsibility, technical information and knowledge, and technical and practical skills) and job performance at the individual, technical, and general levels. Also, the agents' capabilities have had a positive and significant influence on their job performance. It can be concluded that in-service training can influence the agents' capabilities and job performance and improve organizational performance during the COVID-19 pandemic. Thus, the enhancement of in-service training courses' quantity and quality during the COVID-19 pandemic can influence the job performance of the agents at the individual, general, and technical performance levels.

## 1. Introduction

The survival, persistence, and progress of any society depend on the efficiency and quality of the services provided by its institutions and organizations. All countries need competent, motivated, and capable managers and employees as key pillars of organizations to meet the diverse and variable needs of the people and satisfy public welfare. A challenge presently faced by organizations is how they can empower their employees and prepare them to encounter crises and conflicts, satisfy their personal needs, preserve solidarity, and reinforce common aspirations. Managers and employees that cannot solve urgent and organizational problems will miss the opportunities for progress and promotion of themselves and their organizations. To tackle these uncertain, complicated, and dynamic conditions, the only way is to empower the organization, managers, and employees through the acquisition of knowledge and skills, which quickly become obsolete (1, 2). In this regard, a key activity is planning, implementing, and evaluating in-service training courses. Unfortunately, the COVID-19 pandemic has posed in-service training and education of rural institutions to many problems. Following the spread of the COVID-19 pandemic, most institutions all over the world such as the Farmers, Rural People, and Nomads Social Insurance Fund (hereafter the Fund) have suspended training programs and shifted toward distance learning programs. In this regard, in-service training programs for the agents have been dramatically affected as they require hands-on interaction with farmers, rural people, and nomads which originally takes place in rural areas. Many disciplines reported the burden the COVID-19 pandemic has placed on agents e.g. lack of in-service training and failure to consider the skills of staff in new work tasks (3–11). Many institutions have reported difficulty in fulfilling training requirements for a range of different staff during the COVID-19 pandemic. These difficulties have accelerated the development of online training environments and in some cases led to a reduction in conventional lectures within theoretical-based teaching.

In-service training is one of the most effective factors for improving organizational affairs and enhancing efficiency. In-service training has a special place in different organizations as it brings about deeper insights, richer knowledge, and stronger capability and skill in human resources. In-service training can have various achievements, one of which is enabling the trainees so that they can put their efforts as active and independent employees on organizational goals (12). In-service training organizationally refers to training that takes place after a person's recruitment in the organization and aims to prepare people for better fulfillment of their tasks or to improve their capabilities and skills (13, 14). As well, it acts toward enhancing knowledge and awareness, as well as technical, professional, and vocational skills, and triggering desirable behavior in the employees of an organization so that they are prepared to fulfill their job duties and responsibilities (15, 16). Employees should acquire skills in problem-solving and should be enabled to apply the knowledge they have acquired in any possible way. An effective educational system makes its human resource efficient by increasing its career and personal capabilities and skills. If an educational system is not efficient and not tailored to business requirements, the organization will fail in its efforts to enhance the capability of its human resource compared to countries where more optimal conditions are available (17, 18). Knight and Yorke (19) regard career capabilities as a synergic combination of personal qualities and competencies, technical and process skills, and key skills and competencies. According to Aldaihani (20), a capability is the information, skills, and competencies that increase the potential of an individual to take a job, promote, and develop in it, face possible changes in the job, and take another job if he/she wants to change his/her job at different periods of the life. He argues that job capabilities include technical and practical skills, communication and team-working skills, creativity and problem-solving, commitment and responsibility, and information and technical knowledge.

As was explained, in-service training courses should lead to the enhancement of employees' capabilities and job performance. So, one of the most important organizational goals is to increase employees' job performance. Job performance refers to fulfilling the tasks assigned to the human resource by the organization (21). It also encompasses what people do in an organization, how people influence organizational performance (22), and the behavior that is toward achieving measured or valuated organizational goals (23). Job performance is one of the variables that draw much attention in developed countries. For psychologists, job performance is the result of human behaviors. They argue that motivations and needs influence people's performance and finally economic growth and development. Also, it is believed that job performance is a composite construct by which successful employees can be distinguished from unsuccessful employees based on certain criteria. Golparvar and Khaksar (24) argue that employees' job performance should be studied at three levels—personal, technical, and general performance.

According to Ardlç and Işleyen (25), about half of all inequalities observed in jobs are related to not holding effective in-service training courses. So, these courses are faced with shortcomings that reduce their effectiveness. Based on the literature, employees' training in organizations is not at an optimal level. A research study revealed that most experts had not evaluated whether the courses were based on their job requirements and argued that the courses were not practical (26). If the human resource of an organization attends in-service training courses, their empowerment and the efficiency and effectiveness of the organization will be influenced significantly (27). Since a major problem in Iranian organizations is inattention to employees' empowerment and this reduces their job performance and organizational effectiveness and results in the employees' dissatisfaction, in-service training courses are the most important approach to solving these problems.

The farmers, rural people, and nomads social insurance agents (hereafter social insurance agents) are no exception. These people who are in contact with rural people need continuous training to update their skills and knowledge. If agents do not learn new knowledge and enhance their skills, their capability to expand social insurance services among rural people and users in the agricultural sector and among nomads will gradually decrease. Therefore, in-service training courses can improve the agents' capacity, capability, knowledge, and skills. In Iran, the Fund agents highlighted that in-service training and education support of staff was a priority during the COVID-19 pandemic to maintain a continued workforce and future supply line. However, there is limited information investigating the effect of in-service training during the COVID-19 pandemic on the capabilities and job performance of social insurance agents and how best to approach high job performance in the event of future waves of COVID-19 or subsequent pandemics.

This research informs educational planners about the status of in-service training courses and informs officials about the agents' capabilities and job performance and the effect of in-service training on their capabilities and job performance during the COVID-19 pandemic so that they can examine the quantity and quality of the in-service training courses held during the COVID-19 pandemic and improve them. Given the significance of this issue and inattention to in-service training in Iranian organizations, including social insurance agencies of farmers, rural people, and nomads, as well as inattention to the issue by researchers, the present study aimed to explore the effect of in-service training on the capabilities and job performance of social insurance agents in Fars province, Iran.

## 2. Literature review

In a study on errorless training and its effect on employees' job performance, Kern et al. (28) found that this training could be implemented as in-service training and it would finally have a significant effect on improving employees' job performance. Mohaghegh et al. (29) studied virtual in-service training from the perspective of employees. They revealed that the employees who attended virtual in-service training courses were satisfied with the course length and the productivity of the topics discussed in the courses. A research study by Tetteh et al. (30) showed that job training with an emphasis on learning and leadership-based technology had a significant positive effect on employees' performance. A study on the effect of training on organizational efficiency by Niemi (31) shows that training and development are necessary for all organizations, especially for unspecialized and less experienced employees. This study suggests that the performance of the studied organization was improved considering the educational methods and instruments used in it. So, training has had a positive effect on employees' performance, skills, and job efficiency.

Asiamah et al. (32) state that in-service training is one of the best ways to improve job performance. They conclude that in-service training, continuing education, and prolonged tenure are some ways to improve employees' performance. Mundingsari et al. (33) found that people's capabilities had effects on their job performance. Iqbal et al. (34) studied the effect of in-service training on the job performance of technical and vocational teachers. They showed that in-service training had a significant effect on the teachers' performance. According to Suswati et al. (35), in-service training significantly improved people's productivity by 77 percent. Hashim et al. (36) reported that most employees exhibited performance improvement after in-service training. Haryono et al. (37) concluded that the supply of opportunities for the regular attendance of employees in training courses had positive effects on organizational performance. Indeed, job motivation increases with job promotion and training, which will improve job performance. Lyons (38) and Djastuti et al. (39) also conclude that in-service training plays a significant role in enhancing the job performance of employees.

**Figure 1:**
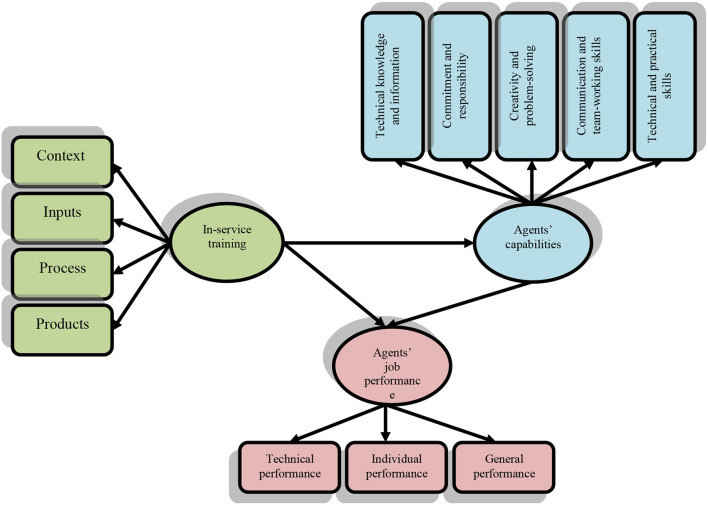
The conceptual framework of the study.

Previous studies have not addressed the effect of in-service training during the COVID-19 pandemic on the capabilities and job performance of social insurance agents. They have mostly investigated the effect of in-service training before the COVID-19 pandemic, and this study can determine the impact of the COVID-19 pandemic on the competencies and job performance of social insurance agents. All governmental and non-governmental organizations need specialized, experienced, and educated people to achieve their goals and missions in critical conditions like the COVID-19 pandemic. To prepare such people, in addition to academic and long-term education, in-service training can enable the organization to supply efficient and specialized human resources in the short run. According to what was discussed above, it is observed that there is a logical relationship between in-service training and employees' capabilities and job performance so that social insurance agents can acquire or improve their knowledge, attitudes, and skills by attending in-service training courses. So, these courses will improve their capabilities and job performance whereas their capabilities will have a direct impact on their job performance. Providing social insurance agents with in-service training will improve their knowledge, attitude, and skills and will help them to be better aware of their own capabilities and be ready for the development and promotion of social insurance in rural communities. This will also improve their job performance. As such, the conceptual framework of the study was developed as presented in Figure 1. According to this framework, in-service training has both direct and indirect effects on agents' job performance. In-service training is assessed by four dimensions of context, input, process, and product. Job performance is also examined at three levels of general, individual, and technical performance. Also, capabilities include technical and practical skills, communication and team-working skills, creativity and problem-solving, commitment and responsibility, and technical information and knowledge. Finally, the research addressed the following hypotheses:

Hypothesis 1: In-service training during the COVID-19 pandemic has significant and positive effects on the capabilities of social insurance agents in Fars province.
Hypothesis 2: In-service training during the COVID-19 pandemic has significant and positive effects on the job performance of social insurance agents in Fars province.
Hypothesis 3: The capabilities of social insurance agents in Fars province have a positive and significant effect on their job performance.

## 3. Methodology

The research is temporally retrospective since data were collected about events that had happened in the past. It is also a quantitative study in terms of paradigm and a non-experimental study in terms of variable control. Also, in terms of statistical operation, it is “causal-relational” and “descriptive-correlational” in which the survey technique was employed for data collection. The research is also an applied study in terms of goal as its results can be used by planners and officials of farmers, rural people, and nomads social insurance funds. The statistical population was composed of all social insurance agents who had attended at least one in-service training course during the COVID-19 pandemic. There were 197 agents across Fars province, and they constituted the research population. The sample, whose size was determined by Krejcie and Morgan's (40) table to be 127 based on the statistical population size, was taken by simple random sampling. This was done by the random number method. In the random number method, we assigned every agent a number. By using a random number table, we then randomly picked a subset of the population.

Fars province has 36 counties, 94 districts, and 113 cities. Its population is 4,851,274 people of whom 3,401,675 people (70.1%) live in urban areas and 1,432,355 people (29.5) reside in rural areas. Over 72 percent of the insured population in the province are members of the production and rural cooperatives, 10.7 percent are members of new institutions, 5.1 percent are members of nomad cooperatives, 7.6 percent are members in *Dehyari*'s, and the remaining are members in state service call centers, post banks, and agricultural joint-stock companies. The total insured population of the province is 145,846 people and the rate of accomplishment to compulsory quota was 73.8 percent based on insurance extension this year. This province has a higher rate of growth in the number of insured people in Iran. It also has a higher number of agents than the other provinces. In the Fund, most in-service training activities were changed to online learning during the COVID-19 pandemic. To cope with the emergency, virtual training environments were arranged, which allowed distance learning activities to continue.

Data were collected with a standard questionnaire. The questionnaire designed in this research was composed of four sections including a section for in-service training courses (41), a section for job performance (24), a section for the agents' capabilities (42), and a section for the agents' demographic characteristics. Regarding in-service training courses, their context, input, process, and product were studied. The assessment of the context aimed to provide a logical context to determine educational goals. At this step, analytical efforts were made to determine the elements of the educational environment and identify the problems, needs, and opportunities within an educational context or situation. The input assessment focused on financial and human resources, policies, training strategies, and barriers and limitations of the educational system in achieving pre-determined goals. The process assessment aimed to recognize or predict executive problems of educational activities and the suitability of the process of fulfilling these activities. The product assessment was performed to determine the extent to which the program goals have been achieved for which goal accomplishment assessment instruments were developed and employed (43).

The agents' job performance was examined at three levels of individual, technical, and general performance. Their capabilities were also assessed in terms of technical and practical skills, communication and team-working skills, creativity and problem-solving, commitment and responsibility, and technical information and knowledge. The research variables were assessed on a five-point Likert scale (from very low to very high). A panel of experts and academic teachers confirmed the face and content validity of the questionnaire and its diagnostic validity was confirmed using the average variance extracted (AVE). To confirm the reliability of the research instrument, a pilot study was conducted in which 30 questionnaires were filled by agents outside the statistical sample and Cronbach's alpha and composite reliability (CR) were calculated (Table 1). The data were collected through face-to-face interviews with the social insurance agents. Thus, 127 agents completed the questionnaire. Data were analyzed in the SPSS23 and AMOS23 software packages. The conceptual framework of the study was validated by confirmatory factor analysis. Also, the status of in-service training courses, agents' capabilities, and their job performance was compared by a one-sample *t*-test. Structural equation modeling was also employed to explore the components of in-service training courses, agents' capabilities, and their job performance.

**Table 1:** The coefficient of measurement and significance level of confirmatory factor analysis and the validity and reliability of the variables.

**Latent**	**variables**	**Observed variables**	**Standardized loading**	**AVE**	**CR**	**α**	***T*-value**
CIPP	Process	Combining theoretical and practical topics (proc6)	0.821	0.51	0.86	0.73	Fixed
		Instructors' mastery of classroom management (proc5)	0.754				6.929
		Instructors' ability to convey educational content (proc4)	0.659				5.959
		Appropriateness of evaluation methods of the teaching and learning process (proc3)	0.648				5.887
		Appropriateness and variety of teaching methods used by teachers (proc2)	0.703				6.247
		Appropriateness of the relationship between the agents and the instructor (proc1)	0.69				6.166
	Context	Relation of the materials presented in the trainings to each other (cont1)	0.787	0.52	0.81	0.75	Fixed
		Attention to the educational-professional needs of agents in designing goals (cont2)	0.738				8.245
		Appropriateness of the provided educational content to the agents' job needs (cont3)	0.664				7.337
		Interest in participating in training courses (cont4)	0.713				7.407
		Up-to-date content presented in the training courses (cont5)	Dropped				-
	Product	Applicability of skills acquired from training courses in the context of job needs (pro1)	0.703	0.54	0.85	0.89	Fixed
		Satisfaction with the experience of participating in social insurance training courses (pro2)	0.745				7.643
		Achievement of educational goals (pro3)	0.823				8.34
		Applicability of knowledge gained from training courses in the context of job needs (pro4)	0.772				7.896
		Change in attitude toward working in social insurance agency after training courses (pro5)	0.642				6.647
	Input	Motivation to participate in social security training courses (inp7)	0.659	0.50	0.87	0.86	Fixed
		Appropriateness of educational space (inp6)	0.774				7.515
		Availability of educational resources (inp5)	0.735				7.201
		Instructors' sufficient teaching skills (inp4)	0.673				6.686
		Availability of educational resources (inp3)	0.842				8.028
		Appropriateness of educational space (inp2)	0.659				6.559
		Motivation to participate in social insurance-related training courses (inp1)	0.551				5.603
	Communication and team-working skills	Sacrifice in solving problems with colleagues (s.com1)	0.675	0.53	0.87	0.86	Fixed
		Interest in creating a suitable environment for conveying information and experiences to colleagues (s.com2)	0.811				7.897
		Reaction with reflection after listening to job tips and tricks (s.com3)	0.763				7.512
		Reflecting facts in providing feedback on workplace issues (s.com4)	0.697				6.949
		Coordination and integration with other partners (s.com5)	0.739				7.312
		Using colleagues' ideas to find the right solution to work problems (s.com6)	0.674				6.742
	Creativity and problem-solving skills	Suggesting new and effective ways to perform job tasks (s.cret1)	0.669	0.50	0.85	0.75	Fixed
		Solving work problems by collecting and analyzing logical information and documentation (s.cret2)	0.711				6.82
		Interest in experimenting and gaining new work experience (s.cret3)	0.778				7.38
		Examining different aspects of the problem to solve work problems (s.cret4)	0.732				6.984
		Making correct and logical inferences and finding correct solutions for work problems (s.cret5)	0.734				6.998
		Taking advantage of opportunities to make positive changes in work practices (s.cret6)	0.635				6.203
	Commitment and responsibility	Positive attitude toward the work and feeling proud of working in it (s.comit5)	0.645	0.50	0.82	0.84	Fixed
		Timely attendance at work and avoidance of absence (s.comit4)	0.598				5.802
		Putting more efforts on maintaining values and implementing the organization's policies and goals (s.comit3)	0.862				7.615
		Active participation in training courses to increase professional skills (s.comit2)	0.752				6.979
		Performing tasks correctly without the need for supervision (s.comit1)	0.634				6.095
	Technical knowledge and information	General information and knowledge in the field of social insurance for farmers, rural people, and nomads (s.info1)	0.739	0.53	0.82	0.84	Fixed
		Feeling the need to attend training courses to improve technical knowledge (s.info2)	0.765				7.291
		Information and knowledge about social security guidelines and instructions (s.info3)	0.700				7.007
		Achieving the goals of the farmers, rural people, and nomads social insurance fund due to the promotion of technical knowledge (s.info4)	0.746				7.391
	Technical and practical skills	Need to attend training courses to perform the assigned tasks correctly (s.tech 1)	0.546	0.50	0.79	0.75	Fixed
		Performing tasks assigned in less time and with better quality with technical knowledge and skills (s.tech2)	0.820				5.834
		Improving my technical and practical skills in performing the assigned tasks (s.tech3)	0.726				5.553
		Saving energy and capital of the social insurance fund due to my technical knowledge and skills (s.tech4)	0.683				5.373
	General performance	Respect for colleagues and respect for their rights and a sense of cooperation toward them (p.gen 1)	0.737	0.67	0.91	0.87	Fixed
		Observance of administrative discipline and regulations (p.gen 2)	0.788				8.985
		Respectful behavior with clients and applicants and efforts to solve their problems (p.gen 3)	0.908				10.497
		Taking care of work tools and saving on their consumption (p.gen 4)	0.924				10.683
		Avoiding time wastage and doing useless things (p.gen 5)	0.744				8.441
	Individual performance	Acknowledging own mistakes in the workplace (p.ind1)	0.527	0.66	0.88	0.92	Fixed
		Feeling responsible for what I have accepted and its consequences (p.ind2)	0.900				6.414
		Working honestly without superior supervision (p.ind3)	0.924				6.476
		Self-sacrifice in times of urgency or human issues (p.ind4)	0.853				6.269
	Technical performance	Attempts to keep the job secrets (p.tech6)	0.813	0.59	0.89	0.87	Fixed
		Being serious about work and maintaining its value (p.tech5)	0.805				10.167
		Follow the work I have undertaken to get the result (p.tech4)	0.811				10.261
		Attempts to convey your job information to others (p.tech3)	0.770				9.569
		Efforts to increase job knowledge (p.tech2)	0.720				8.763
		Compassion for work and trying to provide quality work (p.tech1)	0.685				8.234

## 4. Results

### 4.1. Agents' demographic characteristics

The results showed that 71.7 percent of the agents were male and 28.3 percent were female. In terms of education, the agents were divided into five levels of diploma, associate degree, bachelor's degree, master's degree, and Ph.D. degree. It was found that the highest frequency was for agents with bachelor's degrees (76 agents accounting for 61.3% of the agents) and the lowest was for agents with Ph.D. degrees (2 people, 1.65). Those with associate's degrees, master's degrees, and diplomas were in the second, third, and fourth ranks accounting for 15.3, 12.1, and 9.7 percent of the agents, respectively. Regarding the educational field, most agents were graduates of agriculture (41.2%) and accounting (11.8%). The mean age of the agents was 39 years (a standard deviation of 6.7) with, on average, 7.8 years of job experience in social insurance (a standard deviation of 4.5). They had attended, on average, 9.4 in-service training courses during the COVID-19 pandemic (a standard deviation of 2.01).

### 4.2. Means comparison of research variables with mean intervals

A one-sample *t*-test was used to compare the means of the components of the CIPP model, capabilities, and job performance. Based on the results, there was a significant difference (*p* < 0.000) between the mean of all components of the research variables and the mean intervals whereas the higher and lower bounds of the components of context, product, process, input, communication and team-working skills, creativity and problem-solving skills, commitment and responsibility, technical information and knowledge, technical and practical skills, individual performance, technical performance, and general performance were positive. This means that the mean of the community was significantly higher than the mean interval of the variables. In other words, the mean of these variables was significantly higher than the mean level. Other findings are presented in Table 2.

**Table 2:** The comparison of means of the research variables with mean intervals.

**Variable**	**Scale mean**	**Sample mean**	**S.D**	***t***	**Sig**	**Confidence interval**
Context	15	18.96	2.64	16.84	0.000	3.49, 4.42
Product	15	18.18	3.36	10.63	0.000	2.58, 3.77
Input	21	25.99	4.58	12.27	0.000	4.18, 5.79
Process	18	22.1	3.5	13.2	0.000	3.48, 4.71
Communication and team-working skills	18	22.55	3.82	13.42	0.000	3.88, 5.23
Creativity and problem-solving skills	18	22.51	3.51	14.5	0.000	3.9, 5.13
Commitment and responsibility	15	20.09	3.05	18.81	0.000	4.55, 5.63
Technical information and knowledge	12	15.43	2.46	15.72	0.000	3, 3.86
Technical and practical skills	12	15.6	2.43	16.67	0.000	3.17, 4.03
Individual performance	12	15.99	2.72	16.51	0.000	3.51, 4.47
General performance	15	20.7	3.39	18.93	0.000	5.1, 6.29
Technical performance	18	25.07	3.53	22.59	0.000	6.45, 7,69

### 4.3. Exploration of causal model of the effect of in-service training on agents' capabilities and job performance

Structural equation modeling (SEM) was used to analyze the effects of in-service training during the COVID-19 pandemic on social insurance agents' capabilities and job performance. Accordingly, the measurement section of the model was first evaluated to check the validity and reliability of the variables, and then the structural section was evaluated to confirm the theoretical relations of the conceptual framework's variables. Also, the overall fit of the model was checked by different indices to evaluate the consistency and general agreement of the model with empirical data.

The research employed composite reliability (CR) and average variance extracted (AVE) to measure the reliability and validity of the questionnaire, respectively. Constructs whose CR is >0.6 are reliable enough. The closer the CR is to 1, the more reliable it is (44). Also, constructs whose AVE is >0.5 are valid enough (45). To check the validity of a model, the significance extent and level of paths between each latent variable and its indicators should be checked. So, confirmatory factor analysis was used to test the questions as to whether the indicators considered for each construct or latent variable really accounted for it and how precisely the selected indicators could account for and fit the latent variable. It is observed that the indicators used for the measurement of the studied latent variables had a good fit with the factor structure and the theoretical underpinning of the research given the fact that parameters with values of >1.96 are statistically significant (46).

The measurement model of in-service training during the COVID-19 pandemic showed that the components of input (0.81), context (0.78), product (0.76), and process (0.75) had the highest standard coefficients, respectively. Based on the measurement model of the agents' capabilities, the components of communication and team-working skills (0.78), technical knowledge and information (0.70), creativity and problem-solving skills (0.69), commitment and responsibility (0.68), and technical and practical skills (0.55) were most influential on the agents, respectively. The agents' job performance included technical, general, and individual performance whose standard coefficients were found to be 0.87, 0.81, and 0.71, respectively.

The conceptual model of the research was assessed by chi-square/degrees of freedom (χ2/df), NFI, IFI, CFI, and RMSEA. Given the values reported for each indicator of the model fit in Table 3, χ2/df was found to be 2.05, reflecting the good fit of the model. To check how well a model performs, especially vs. other possible models, in accounting for a set of observed data, the alternative model examination indicators (NFI, IFI, and CFI) were used. These indicators were estimated at 0.87, 0.93, and 0.93, respectively. Finally, RMSEA was employed to see how a conceptual model combines fitness and saving. The value found for this indicator (0.06) shows the control of the measurement error in the model. Accordingly, most reported indicators had acceptable values for the general fit of the model, so it can be said that the model was generally consistent with the data used.

**Table 3:** The model fitness indicators.

**Test**	**Recommended value**	**Proposed model**
Likelihood ratio Chi-square (x2)	Insignificant x2 (*p* > 0.05)	0.000
Normed chi-square (x2/df)	x2/df < 5	2.05
Root mean squared error	RMSEA < 0.08	0.06
Normed fit index	NFI > 0.9	0.87
Incremental fit index	IFI = Values close to 1	0.93
Comparative fit index	CFI > 0.9	0.93

**Figure 2:**
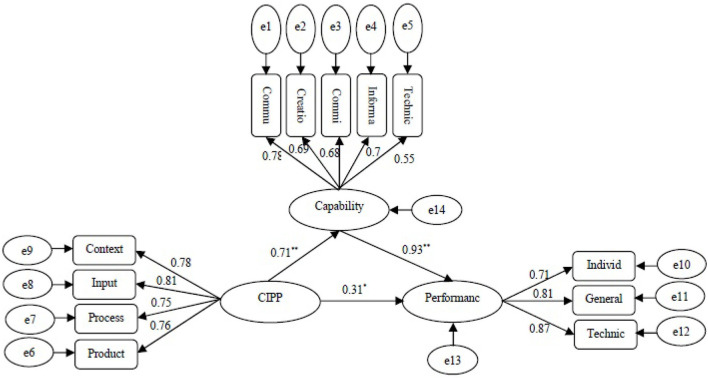
The structural model of the research.

Based on the structural model of the research, in-service training during the COVID-19 pandemic has had a direct impact on the agents' capabilities and a direct and indirect impact on their job performance. The agents' capabilities have a direct impact on their job performance. The results show that the effect of in-service training of the Fund during the COVID-19 pandemic has been positive and significant on the agents' capabilities (β = 0.71, *P* < 0.000). This supports hypothesis 1, which holds that in-service training during the COVID-19 pandemic has significant and positive effects on the capabilities of social insurance agents in Fars province. It was revealed that the effect of in-service training during the COVID-19 pandemic was positive and significant on the agents' job performance (β = 0.31, *P* < 0.05). So, hypothesis 2, i.e., in-service training during the COVID-19 pandemic has significant and positive effects on the job performance of social insurance agents in Fars province, is confirmed. Finally, the agents' capabilities had a significant and positive impact on their job performance (β = 0.42, *P* < 0.05). So, hypothesis 3, i.e., the capabilities of social insurance agents in Fars province have a positive and significant effect on their job performance, is confirmed (Figure 2).

Based on the results, the coefficient of determination (*R*^2^) is 0.517 for the agents' capabilities. This means that 51.7 percent of the agents' capabilities of social insurance fund in Fars province is related to in-service training. Also, *R*^2^ was estimated at 0.548 for the agents' job performance. So, in-service training and the agents' capabilities can predict 54.8 percent of the variance in the variable of the agents' job performance.

## 5. Discussion

The results show that in-service training during the COVID-19 pandemic has a significant effect on the capabilities of social insurance agents. In other words, if the components of an in-service training course (context, input, process, and product) are formulated correctly, they can promote the agents' information, skills, and competencies in their job. Also, in-service training during the COVID-19 pandemic empowers the agents in the face of likely changes in their job or their decision to change their careers in the future. Other researchers (47–52) have also concluded that in-service training can enhance the employees' capabilities in different aspects. They argue that in-service training can increase the employees' technical and practical skills, communication and team-working skills, creativity and problem-solving skills, commitment and responsibility, and technical information and knowledge.

Based on the findings, if in-service training during the COVID-19 pandemic aims to prepare the agents for taking over their responsibilities and improve their capabilities and skills, they will fulfill the tasks assigned by the organization as well as possible. This is consistent with the reports of Dehghani et al. (53), Rabiee et al. (54), Tanjung et al. (55), Asiamah et al. (32), Djastuti et al. (39), Haryono et al. (37), Hashim et al. (36), Iqbal et al. (34), Lyons (38), and Mundingsari et al. (33), who have concluded that in-service training influences the employees' job performance at the individual, general, and technical levels. In other words, the more extensively the in-service training courses cover the skills required by the job, the more consistent the behaviors of the employees will be with the achievement of the organizational goals and the promotion of job performance.

The results reveal that stronger technical and practical capabilities, communication and team-working skills, creativity and problem-solving skills, commitment and responsibility, and technical knowledge and information of agents are effective in enhancing their job performance. In other words, the agents' capabilities are the result of their organizational activities and their effectiveness and efficiency are enhanced as components of their job performance. Similarly, Dehghani et al. (53), Inwood (56), Bieńkowska and Tworek (57), Mundingsari et al. (33), Ren et al. (58), and Udin et al. (59) state that the more capable the employees are, the more likely their job performance enhance. They have concluded that employees' empowerment in any possible way can have plenty of outputs, the most important ones being the enhancement of their job performance and the achievement of short-term and long-term organizational goals. In other words, there is a mutual relationship between employees' capabilities and their performance.

## 5. Conclusion

The COVID-19 pandemic afforded an opportunity to study the content and process of change during an active crisis. In this case of in-service training, our findings provide insight into the ways an in-service training system adapts to unanticipated circumstances. In this research, we first reviewed the literature on in-service training, empowerment and capabilities of social insurance agents, their job performance, and the appraisal of in-service training courses during the COVID-19 pandemic. The review of the literature and theoretical framework revealed the logical and theoretical relationship of in-service training with employees' capabilities and job performance. Then, the in-service training courses during the COVID-19 pandemic were assessed from the perspective of the agents at four levels of the context, inputs, educational process, and products of the in-service training courses. The results revealed that the means of all four levels were higher than the mean intervals. It can, therefore, be concluded that the in-service training courses during the COVID-19 pandemic held were at an optimal level of quality. In other words, the social insurance agents believe that the goals and programs, educational content, duties and responsibilities of the attendees (context dimension), proper plan to accomplish educational goals, implementation requirements, human resource educational program, resource and facility requirements (input dimension), the implementation of the developed program and the different steps of the in-service training program (process dimension), and products and efficiency of in-service training (product dimensions) have been planned and fulfilled properly. Also, the results showed that the mean capabilities of the agents were significantly higher than the mean intervals. So, it can be concluded that social insurance agents were highly capable in communication and team-working skills, creativity and problem-solving skills, commitment and responsibility, technical information and knowledge, and technical and practical skills so that the agents can improve and develop their capabilities in different career fields. In addition, the mean job performance of the agents at the individual, general, and technical levels was higher than the mean intervals. In general, it can be argued that the social insurance agents in Fars province have appropriate job performance despite the limited number of in-service training courses held for them during the COVID-19 pandemic.

At the end of the research, the conceptual framework of the research, which would show the effect of in-service training during the COVID-19 pandemic on the agents' capabilities and job performance, was assessed. Based on the results, in-service training during the COVID-19 pandemic has a significant and positive effect on the social insurance agents' capabilities and job performance. This means that a well-designed in-service training course (in terms of context, inputs, process, and products) during the COVID-19 pandemic can improve the agents' capabilities in communication and team-working skills, creativity and problem-solving skills, commitment and responsibility, technical information and knowledge, and technical and practical skills. In addition, the enhancement of in-service training courses' quantity and quality during the COVID-19 pandemic can influence the job performance of the agents at the individual, general, and technical performance levels. On the other hand, the agents' capabilities had a significant and positive effect on their job performance. This implies that the more capable the social insurance agents are, the higher their job performance is. Thus, it can be concluded that in-service training during the COVID-19 pandemic can enhance the job performance of social insurance agents both directly and indirectly (by influencing their capabilities).

According to the results, it is recommended to conduct a comprehensive needs assessment of the agents and even educational planning before any attempts to plan and implement in-service training courses. This will allow holding training courses that will be consistent with the employees' needs and the organizational goals and perspectives. In other words, the process of studying educational needs and needs assessment should be investigated scientifically, exclusively, and precisely and it should be validated, for which the experts and agents can be asked to help because it will be the underpinning of the agents' educational process. Obviously, this educational needs assessment (at three analysis levels of the organization, job, and personal) should encompass all educational needs of the agents, including their general and technical educational requirements. To improve the suitability of the input dimension of the courses, it is suggested to provide the agents with adequate information before their attendance in the educational courses using modern information-sharing methods to enhance their motivation and have a clear mentality among them about the courses. Also, experienced teachers and instructors should be recruited to increase the efficiency and productivity of in-service training. To improve the quality of the process dimension, it is recommended to use other teaching and learning methods than lectures, e.g., workshops, problem-solving, and online teaching, to increase the effectiveness and efficiency of the educational program. Teachers can use any of these methods depending on the course and their pros and cons. Furthermore, the course officials and experts should carefully monitor the barriers and implementation problems during the training courses and tackle them. Given the significance of providing the agents with practical knowledge and skills in the courses, it is recommended to the teachers to adjust course contents to educational needs and include practical courses related to the agents' job requirements in order to enhance their knowledge and skills so as to be applicable in practice.

Similar to most studies, there were main limitations in this study. The first limitation is related to the methodology applied in the study. In this research, only the questionnaire and cross-sectional survey were used for data collection. However, the examination of in-service training during the COVID-19 pandemic using qualitative methods such as in-depth interviews, grounded theory, case studies, mixed methods, and so forth may provide more realistic insights into their aspects and determinants. The second limitation is related to the sample size used in the study. Although the size of the sample used in this study was completely sufficient (based on the sampling table proposed by Krejcie and Morgan), future researchers can use a larger sample size and choose more provinces. This can increase the robustness and generalization capability of the results.

## Data availability statement

The raw data supporting the conclusions of this article will be made available by the authors, without undue reservation.

## Ethics statement

Ethical review and approval was not required for the study on human participants in accordance with the local legislation and institutional requirements. Written informed consent from the participants was not required to participate in this study in accordance with the national legislation and the institutional requirements.

## Author contributions

Conceptualization, methodology, and software: HG and PA. Analyzing: PA. Writing and revising: HG, AM, CB, and PA. All authors contributed to the article and approved the submitted version.
